# Retinal Neuroprotective Effects of Flibanserin, an FDA-Approved Dual Serotonin Receptor Agonist-Antagonist

**DOI:** 10.1371/journal.pone.0159776

**Published:** 2016-07-22

**Authors:** Aaron S. Coyner, Renee C. Ryals, Cristy A. Ku, Cody M. Fischer, Rachel C. Patel, Shreya Datta, Paul Yang, Yuquan Wen, René Hen, Mark E. Pennesi

**Affiliations:** 1 Casey Eye Institute, Oregon Health & Science University, Portland, Oregon, United States of America; 2 Baylor University Medical Center, Dallas, Texas, United States of America; 3 New York State Psychiatric Institute, Columbia University, New York, New York, United States of America; University of Cologne, GERMANY

## Abstract

**Purpose:**

To assess the neuroprotective effects of flibanserin (formerly BIMT-17), a dual 5-HT_1A_ agonist and 5-HT_2A_ antagonist, in a light-induced retinopathy model.

**Methods:**

Albino BALB/c mice were injected intraperitoneally with either vehicle or increasing doses of flibanserin ranging from 0.75 to 15 mg/kg flibanserin. To assess 5-HT_1A_-mediated effects, BALB/c mice were injected with 10 mg/kg WAY 100635, a 5-HT_1A_ antagonist, prior to 6 mg/kg flibanserin and 5-HT_1A_ knockout mice were injected with 6 mg/kg flibanserin. Injections were administered once immediately prior to light exposure or over the course of five days. Light exposure lasted for one hour at an intensity of 10,000 lux. Retinal structure was assessed using spectral domain optical coherence tomography and retinal function was assessed using electroretinography. To investigate the mechanisms of flibanserin-mediated neuroprotection, gene expression, measured by RT-qPCR, was assessed following five days of daily 15 mg/kg flibanserin injections.

**Results:**

A five-day treatment regimen of 3 to 15 mg/kg of flibanserin significantly preserved outer retinal structure and function in a dose-dependent manner. Additionally, a single-day treatment regimen of 6 to 15 mg/kg of flibanserin still provided significant protection. The action of flibanserin was hindered by the 5-HT_1A_ antagonist, WAY 100635, and was not effective in 5-HT_1A_ knockout mice. *Creb*, *c-Jun*, *c-Fos*, *Bcl-2*, *Cast1*, *Nqo1*, *Sod1*, and *Cat* were significantly increased in flibanserin-injected mice versus vehicle-injected mice.

**Conclusions:**

Intraperitoneal delivery of flibanserin in a light-induced retinopathy mouse model provides retinal neuroprotection. Mechanistic data suggests that this effect is mediated through 5-HT_1A_ receptors and that flibanserin augments the expression of genes capable of reducing mitochondrial dysfunction and oxidative stress. Since flibanserin is already FDA-approved for other indications, the potential to repurpose this drug for treating retinal degenerations merits further investigation.

## Introduction

Inherited retinal dystrophies (IRDs), such as retinitis pigmentosa, are clinically and genetically diverse, and account for approximately 5% of the vision loss in the Western world. [1–3] There are no effective treatments for most of these disorders, but gene therapy shows promise for a small subset of IRDs. [4–8] However, with almost 180 genes and thousands of distinct mutations, developing gene replacement strategies for all IRD patients will be a long and difficult endeavor. [2] The development of gene-independent therapies is therefore essential in preserving retinal morphology and function until suitable gene therapies can be developed. Additionally, synergistic rescue may be achieved with neuroprotective agents in combination with gene or cell-based therapies.

The neurotransmitter, serotonin (5-hydroxytryptamine or 5-HT), has been shown to modulate retinal processing, although the full extent of the role of serotonin in the retina remains unclear. [9–20] 5-HT receptors have been sub-divided into seven major classes (5-HT_1-7_) based on structural, functional and pharmacological criteria, with distinct molecular properties further dividing these classes into over 17 different subtypes. [14, 21] Chen et al. demonstrated evidence for gene expression of 5-HT receptors in the mammalian retina using quantitative transcriptome analysis. [22] Localization studies expressing eGFP under the endogenous 5-HT receptor promoter suggest the presence of 5-HT_2A_ and 5-HT_3A_ receptors in bipolar cells,[23, 24] and 5-HT_2A_ in photoreceptor terminals. [25] Despite evidence of serotonin and 5-HT receptors in the mammalian retina, their likely low expression in the retina has hindered exact localization and evaluation of function. [22]

Recent studies conducted by our lab and others suggest that certain serotonin receptor agonists and antagonists provide neuroprotection against light-induced retinopathy. [15–20, 26] 5-HT_1A_ receptor agonists, such as 8-OH-DPAT and AL-8309B, provided anti-oxidant protection against light-damage in albino rats and in a genetic mouse model for AMD. [15–17] 5-HT_2A_ receptor antagonists, ketanserin and sarpogrelate, protected against light-induced retinopathy in albino Balb/c mice and in the Stargardt *Abca4*^-/-^*Rdh8*^-/-^ mouse model. [20, 26] In addition, simultaneous treatment with a 5-HT_1A_ agonist and a 5-HT_2A_ antagonist has shown an additive neuroprotective effect. [20, 22, 27] Altogether, these reports suggest that 5-HT_1A_ receptor agonists and 5-HT_2A_ receptor antagonists can elicit neuroprotective effects in the retina and warrant their continued investigation as a therapeutic class in treatment of retinal degeneration.

Flibanserin, marketed as Addyi^™^ (Sprout Pharmaceuticals), was recently approved by the Food and Drug Administration (FDA) for the treatment of hypoactive sexual desire disorder (HSDD). [28–33] Seven phase III clinical trials demonstrated that a daily 100 mg dose of flibanserin was safe and resulted in an improvement of HSDD-related symptoms in pre-menopausal women. [30–33] In CHO cells, flibanserin has a high affinity for serotonergic 5-HT_1A_ and 5-HT_2A_, as well as dopaminergic D_4_ receptors, although D_4_ receptor binding has yet to be verified in human brain tissue. [34–39]

The determination of retinal protection and cell survival mechanisms conferred by neuroprotective agents, such as flibanserin, is aided by evaluation in models with widely-described cell death mechanisms. The relatively synchronous and rapid progression of apoptosis in light-induced retinopathy has provided many insights into the mechanisms of cell death. [40] Therefore, to evaluate the neuroprotective potential of flibanserin and its mechanism of action, we tested it in the well-established light-induced retinopathy model. [20, 41–45]

## Methods

### Animals

All experiments and animal handling procedures were approved by and performed in compliance with the policies of the Institutional Animal Care and Use Committee at Oregon Health & Science University (IACUC Protocol Number IS03147) and adhered to the ARVO Statement for the Use of Animals in Ophthalmic and Vision Research. Albino BALB/cJ mice were purchased from The Jackson Laboratory (Bar Harbor, ME). 5-HT_1A_^-/-^ mice (5-HT_1A_ knockout mice) were generously provided by Dr. René Hen (Columbia University, New York, NY). [46] 5-HT_1A_^-/-^ mice were originally on a pure 129/Sv background, but backcrossed with albino BALB/c mice for nine generations (F9) before being used in experiments. Genotypes were confirmed using PCR. Mice utilized in these studies were males between two and three months old. Mice were housed in a 12-hour alternating light/dark cycle room. The light cycle (~15 lux) occurred from 9:00 PM to 9:00 AM, and dark cycle from 9:00 AM to 9:00 PM. Following data acquisition, all mice were euthanized via gradual CO_2_ inhalation and cervical dislocation.

### PCR Genotyping

PCR analysis was performed using Quick-load Taq (Taq 2x Master Mix, New England Biolabs, Ipswich, MA) and the following primer sequences (Integrated DNA Technologies, San Diego, CA): 5-HT_1A_ promoter (forward): 5’-CAG TCT CTA GAT CCC CTC CCT-3’; 5-HT_1A_ coding sequence (reverse): 5’-GGG CGT CCT CTT GTT CAC GTA-3’; and tTA insert (reverse): 5’-AAG GGC AAA AGT GAG TAT GGT-3’. A 25 μL PCR reaction was performed in a Veriti thermal cycler (Applied Biosystems, Coster City, CA). Each PCR reaction consisted of 12.5 μL Quick-load Taq, 1 μL of primer mix (1 μL of 200 μg/μL 5-HT_1A_ promoter, 0.5 μL of 200 μg/μL 5-HT_1A_ coding sequence, 0.5 μL of 200 μg/μL tTA insert, and 18 μL distilled H_2_O), 1 μL genomic DNA, and 10.5 μL distilled H_2_O. PCR cycle was as follows: initial denaturation for 1 minute at 95°C, then 30 cycles of 95°C for 45 s, 50°C for 30 s, 72°C for 60 s, and a final extension at 72°C for 60 s. No-template DNA controls, wildtype, and 5-HT_1A_ heterozygote and homozygote controls were utilized with each PCR genotyping experiment. PCR products were analyzed via gel electrophoresis using a 1.5% agarose gel ran at 90 mV for 50 minutes. Bands were visualized using EZ-Vision (EZ-Vision Three DNA Dye as Loading Buffer 6X, Amresco, Solon, OH) and a UV transilluminator (GelDoc-It, UVP, Upland, CA). The WT and KO alleles were identified as 600 bp and 300 bp PCR products, respectively.

### Drug Preparation

Flibanserin (Sigma-Aldrich, St. Louis, MO) was dissolved in Kollisolv PEG E 400 (PEG 400) (Sigma-Aldrich, St. Louis, MO), warmed to 60°C and diluted to 35% (v/v) in 0.9% saline (Hospira, Inc., Lake Forest, IL). The vehicle was 35% (v/v) PEG 400 in 0.9% saline. WAY 100635 was dissolved in 0.9% saline. The vehicle for this experiment was 0.9% saline.

### Five-Day Drug Time Course

Two hours into the 12-hour dark cycle, mice were injected intraperitoneally (10 mL/kg) under dim red light. Mice were dosed with vehicle, 0.75, 1.5, 3.0, 6.0, 9.0 or 15 mg/kg flibanserin 48 hours, 24 hours and immediately prior to a one-hour bright light exposure, and 24 and 48 hours after light exposure. Naïve mice were not injected or exposed to bright light.

### One-Day Drug Time Course

Two hours into the 12-hour dark cycle, mice were injected intraperitoneally (10 mL/kg) under dim red light. A single dose of vehicle, 3, 6, 9 or 15 mg/kg of flibanserin was administered immediately before a one-hour bright light exposure in order to evaluate a shortened one-day treatment regimen. To assess 5-HT_1A_-mediated effects, 10 mg/kg WAY 100635, a selective 5-HT_1A_ antagonist (K_i_ = 2),[47] was administered to mice 30 minutes prior to 6 mg/kg flibanserin. Additionally, 5-HT_1A_^-/-^ mice were also administered 6 mg/kg flibanserin immediately prior to bright light exposure. Because WAY 100635 is also a potent D_4_ agonist (K_i_ = 16),[47] 10 mg/kg WAY 100635 was injected alone prior to bright light exposure in order to assess whether a D_4_ agonist, delivered at a concentration similar to that of 15 mg/kg flibanserin, could provide any neuroprotective effects against light-induced retinopathy. Naïve mice were not injected or exposed to bright light.

### Light-Induced Retinopathy

Four compact fluorescent lamps (42 watts, 6500K) were installed in a custom-built light box producing 10,000 lux of uniform light, and capable of holding 16 mice. [20] Two hours into the 12-hour dark cycle, mice were placed into the light box and exposed to bright light for one hour. Temperature and humidity were regulated using a portable air conditioning unit. Mice were monitored throughout light exposure and were given access to water. Vehicle-injected control mice were used in each experiment to ensure the occurrence of light-induced retinopathy. [20]

### In Vivo OCT Imaging

Retinas were imaged as previously reported. [20, 48] Seven days following bright light exposure, mice were sedated using 1.5% isoflurane delivered via a nose cone, corneas were anesthetized with 0.5% proparacaine, and pupils were dilated using a combination of 1% tropicamide and 2.5% phenylephrine. Artificial tears were used to maintain corneal clarity. Mice were seated in a Bioptigen AIM-RAS holder and spectral domain optical coherence tomography (SD-OCT) images were obtained using an Envisu R2200-HR SD-OCT instrument (Bioptigen, Durham, NC). [20, 48] Each eye was imaged using linear horizontal scans in the temporal and nasals quadrants and linear vertical scans in the superior and inferior quadrants.

### Image Processing and Segmentation

SD-OCT scans were processed and segmented as previously reported. [20, 48] Briefly, SD-OCT scans were converted into image files using ImageJ (version 1.48; National Institutes of Health, Bethesda, MD) and, using a custom designed SD-OCT segmentation program built in IGOR Pro (IGOR Pro 6.37; WaveMetrics Inc., Lake Oswego, OR), total retinal (TR) and receptor plus (REC+) thicknesses were measured. [20, 48, 49] The thickness from Bruch’s membrane to the vitreous/retinal nerve fiber layer interface is defined as TR. REC+ is defined as the thickness from Bruch’s membrane to the inner nuclear layer/outer plexiform layer interface. [20, 48, 49]

### Electroretinograms (ERGs)

ERGs were performed as previously reported. [20, 50, 51] ERGs were recorded two to seven days after SD-OCT imaging. Dark-adapted mice were anesthetized with ketamine (100 mg/kg)/xylazine (10 mg/kg), corneas were anesthetized, and pupils were dilated. Mice were placed on a heated platform (37°C) inside of a Ganzfeld dome coated with a highly reflective paint. Platinum signal electrodes were placed on the center of each cornea, and additional electrodes were placed in the forehead and tail as reference and ground, respectively. Finally, a lubricant, Goniovisc, Hypromellose 2.5% (Dynamic Diagnostics, Westland, MI), was applied to the corneas. ERG traces were averaged and analyzed using custom software. Light intensities ranged from -4.34 to 3.55 log cd•s/m^2^. [20, 50, 51] The number of trials at each light intensity was decreased as the intensity of the flashes increased in order to prevent light adaptation. The time between each flash was increased to allow for pigment regeneration. The minimum interstimulus interval was seven seconds, and the maximum was 70 seconds. Flashes were calibrated as previously described. [20, 50, 51]

### RNA Extraction and RT-qPCR

Two hours into the 12-hour dark cycle, mice were injected intraperitoneally (10 mL/kg) under dim red light. Mice were dosed with vehicle or 15 mg/kg flibanserin 48 hours, 24 hours and immediately prior to a one-hour bright light exposure, and 24 and 48 hours after light exposure. Retinas from vehicle-injected mice and flibanserin-injected mice were harvested 48 hours and 72 hours after light exposure. Total RNA was extracted from retinas using an RNeasy Mini Kit (Qiagen, Hilden, Germany). One microgram of total RNA was converted to cDNA using a Bio-Rad iScript cDNA synthesis kit (Hercules, CA). Primers were designed with the Integrated DNA Technologies Realtime PCR Tool (S1 Table, Coralville, Iowa). PCR reaction mixtures consisted of 10 μL DyNAmo HS SYBR green (Thermo Scientific, Waltham, MA), 1 μL cDNA template, 1 μL of 200 μM gene-specific primer sets, and 8 μL distilled water. Using a Bio-Rad Chromo4 System (Hercules, CA), samples were initially denatured for 2 minutes at 95°C, followed by 40 cycles at 95°C for 15 seconds and 58°C for 30 seconds. Gene expression was normalized to β-actin and relative gene expression in flibanserin-injected mice was compared to vehicle-injected mice using the ΔΔCt method.

### Statistical Analysis

Average outer retinal thickness (REC+), total retinal thickness (TR), a-wave amplitudes and b-wave amplitudes were calculated for each animal. Representative topographic distributions were an average of the REC+ thicknesses from the right eyes for all animals in each group, centered at the optic nerve. For scatterplot graphs, left and right eye data was combined for each animal and then averaged for each group. One-way analysis of variance (ANOVA) was used to compare REC+, TR and three different ERG measurements: b_max,rod_ at -2.14 log cd•s/m^2^, and a_max,rod+cone_ and b_max,rod+cone_ at 3.55 log cd•s/m^2^ using Prism (Prism 6.0; GraphPad Software Inc., La Jolla, CA). Multiple t-tests were performed to compare the expression of individual genes in flibanserin- and vehicle-injected mice at 48- and 72-hours after light exposure. For all analyses, a *P* value less than 0.05 was considered significant.

## Results

### Flibanserin-Mediated Neuroprotection

#### Five-day time course

**Fig 1:**
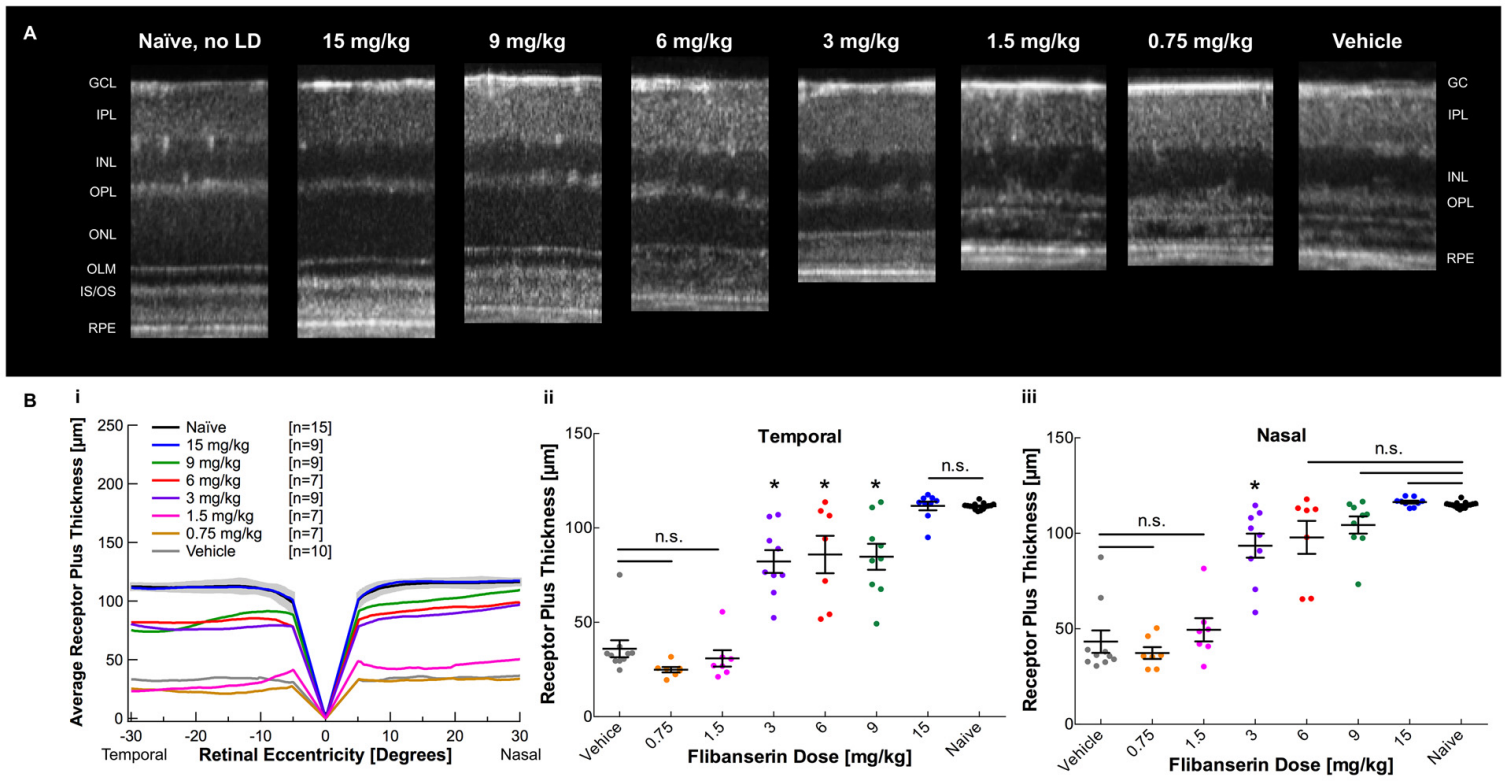
A 5-day time course of flibanserin protects retinal morphology from bright light exposure in a dose-dependent manner. (**A**) Representative linear SD-OCT scans of nasal retina show increasing outer nuclear layer thickness with flibanserin treatment, in a dose-dependent manner. A naïve non-light damaged mouse shows an easily distinguishable OPL, ONL, OLM, IS/OS, and RPE. GCL-ganglion cell layer, IPL-inner plexiform layer, INL-inner nuclear layer, OPL-outer plexiform layer, ONL-outer nuclear layer, OLM-outer limiting membrane, IS/OS-inner segment/outer segment junction, RPE-retinal pigment epithelium. (**B i**) A spider graph of average right-eye receptor plus values demonstrates that flibanserin-mediated protection increases in a dose-dependent manner starting at 3 mg/kg, with a 15 mg/kg dose showing comparable receptor plus thicknesses to naïve (non-light damaged) mice. The gray area indicates ± 2 SD of the naïve averaged data. (**B ii, iii**) Receptor plus thicknesses, with each dot representing average right and left eye thickness from one mouse, demonstrate that 15 mg/kg flibanserin achieves complete morphological protection in the **(B ii)** temporal quadrant and the **(B iii)** nasal quadrant. Group averages are represented as mean ± standard error bar. * indicates a significant difference from both the vehicle-treated group and the naïve group, *P* < 0.05. n.s. indicates non-significance with *P* > 0.05.

Bright light exposure resulted in thinning of the outer nuclear layer (ONL) and obscuration of the inner segment/outer segment junction (IS/OS) in retinas of vehicle-injected mice, and 0.75 and 1.5 mg/kg flibanserin-injected mice, as compared the normal morphology of naïve mice (Fig 1A). Injections with 3, 6, 9, and 15 mg/kg of flibanserin not only preserved the ONL and IS/OS, but also revealed a dose-dependent preservation of ONL thickness (Fig 1A and 1B). Average REC+ thicknesses of 3, 6, 9, and 15 mg/kg flibanserin were significantly increased from vehicle-treated mice (Table 1, Fig 1B), and administration of 15 mg/kg flibanserin provided maximum protection of retinal morphology, resulting in non-significant differences in REC+ thickness as compared to non-light damaged naïve mice.

**Table 1:** Summary of OCT findings.

		Nasal		Temporal	
Group	*n*	TR (μm) Mean ± SD	Rec+ (μm) Mean ± SD	TR (μm) Mean ± SD	Rec+ (μm) Mean ± SD
*Five-day Time Course*					
Vehicle	10	125 ± 18	43 ± 19	117 ± 14	36 ± 14
Flibanserin, 0.75 mg/kg	7	119 ± 8	37 ± 8	106 ± 4	25 ± 4
Flibanserin, 1.5 mg/kg	7	132 ± 16	50 ± 16	113 ± 13	31 ± 11
Flibanserin, 3 mg/kg	9	175 ± 20	94 ± 19	165 ± 19	82 ± 18
Flibanserin, 6 mg/kg	7	179 ± 23	98 ± 23	168 ± 26	86 ± 26
Flibanserin, 9 mg/kg	9	185 ± 14	104 ± 14	166 ± 21	85 ± 21
Flibanserin, 15 mg/kg	9	196 ± 3	116 ± 2	191 ± 7	112 ± 7
Naive	15	195 ± 2	115 ± 2	191 ± 2	112 ± 2
*One-day Time Course*					
Vehicle	7	136 ± 22	52 ± 21	119 ± 18	37 ± 17
Flibanserin, 3 mg/kg	5	120 ± 12	39 ± 11	116 ± 21	36 ± 19
Flibanserin, 6 mg/kg	6	194 ± 3	116 ± 2	192 ± 3	112 ± 2
Flibanserin, 9 mg/kg	6	199 ± 2	120 ± 2	196 ± 2	118 ± 3
Flibanserin, 15 mg/kg	7	197 ± 3	114 ± 2	194 ± 3	111 ± 2
Naive	15	195 ± 2	115 ± 2	191 ± 2	112 ± 2

**Fig 2:**
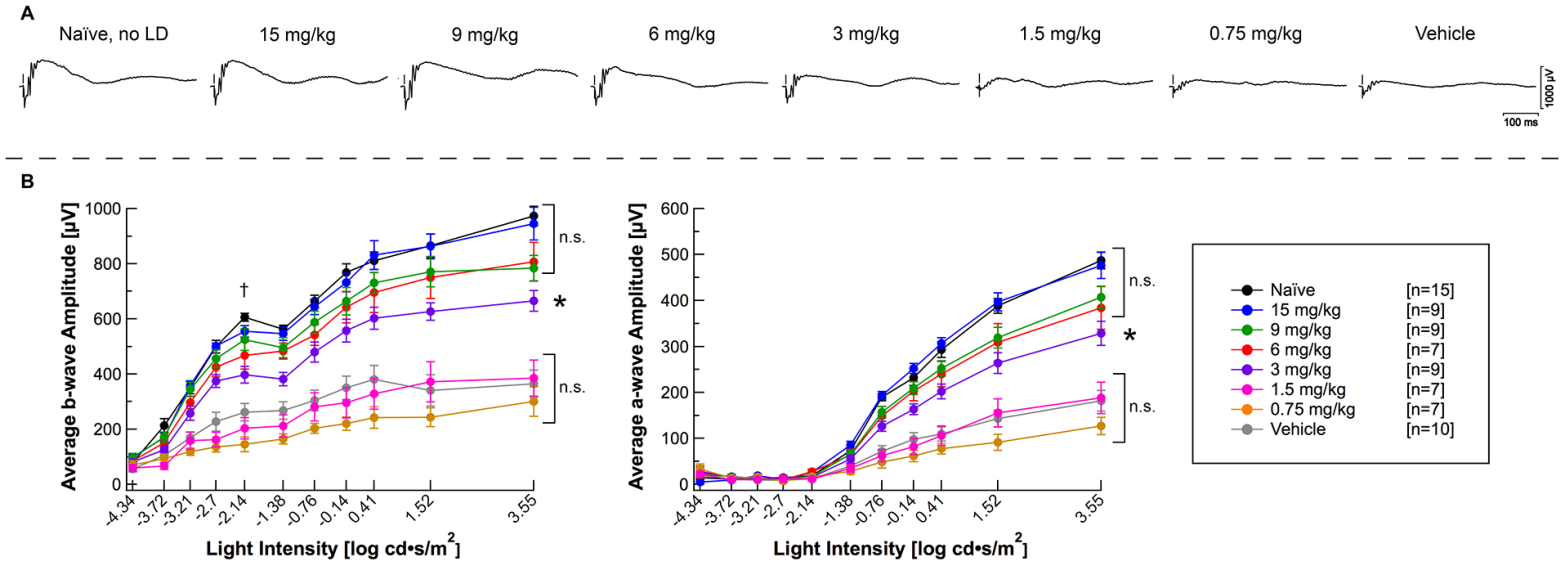
A 5-day time course of flibanserin preserves retinal function in a dose-dependent manner, as measured by ERG. (**A**) Representative ERG traces at the 3.55 log cd•s/m^2^ light intensity demonstrating dose-dependent improvements in ERG responses as compared to vehicle-injected mice. (**B**) Mice treated with doses of 3 mg/kg or greater of flibanserin showed significantly higher ERG b-wave and a-wave responses at the highest ERG flash intensity (3.55 log cds/m^2^) as compared to vehicle-treated mice. Individual ERGs were averaged with ERGs for mice within its respective group and are represented as mean ± standard error. * indicates a significant difference from both the vehicle-treated group and the naïve group, *P* < 0.05. n.s. indicates non-significance with *P* > 0.05. The responses at the ERG b_(max,rod)_ light intensity, indicated by the “†”, were statistically evaluated and are represented in S1A Fig.

ERGs demonstrated severe attenuation of retinal function in vehicle-injected mice versus naïve mice, as observed in the representative ERG waveforms and scotopic a-wave and b-wave amplitudes (Fig 2A and 2B, S1A Fig, Table 2). Intensity response curves and average a- and b-wave amplitudes highlight the variable protection between treatment groups and a general dose-dependent effect (Fig 2B). 15 and 9 mg/kg flibanserin groups were not significantly different from the naïve group in b-wave responses (b_max,rod_, b_max,rod+cone_), or a-wave responses (a_max,rod+cone_) (Fig 2B, S1A Fig, Table 2). The 1.5 and 0.75 mg/kg flibanserin groups failed to preserve retinal function entirely; ERG responses were not significantly increased from vehicle-injected mice. The 3 mg/kg and 6 mg/kg flibanserin-treated mice showed variable protection in ERG responses, but generally showed reduction of responses from naïve mice but improved from vehicle-treated mice.

**Table 2:** Summary of ERG parameters.

Group	*n*	b_max,rod_ (μV) Mean ± SD	a_max,rod+cone_ (μV) Mean ± SD	b_max,rod+cone_ (μV) Mean ± SD
*Five-day Time Course*				
Vehicle	10	262 ± 99	181 ± 71	365 ± 155
Flibanserin, 0.75 mg/kg	7	146 ± 70	127 ± 50	301 ± 142
Flibanserin, 1.5 mg/kg	7	203 ± 104	189 ± 91	386 ± 174
Flibanserin, 3 mg/kg	9	398 ± 91	329 ± 80	666 ± 114
Flibanserin, 6 mg/kg	7	468 ± 131	384 ± 123	807 ± 186
Flibanserin, 9 mg/kg	9	525 ± 118	407 ± 71	785 ± 139
Flibanserin, 15 mg/kg	9	556 ± 64	476 ± 86	945 ± 177
Naive	15	606 ± 58	487 ± 68	973 ± 129
*One-day Time Course*				
Vehicle	7	251 ± 47	229 ± 51	424 ± 101
Flibanserin, 3 mg/kg	5	226 ± 87	167 ± 88	393 ± 167
Flibanserin, 6 mg/kg	5	579 ± 93	466 ± 118	917 ± 187
Flibanserin, 9 mg/kg	6	464 ± 73	357 ± 110	668 ± 284
Flibanserin, 15 mg/kg	7	513 ± 65	459 ± 53	934 ± 121
Naive	15	606 ± 58	487 ± 68	973 ± 129

#### One-day time course

**Fig 3:**
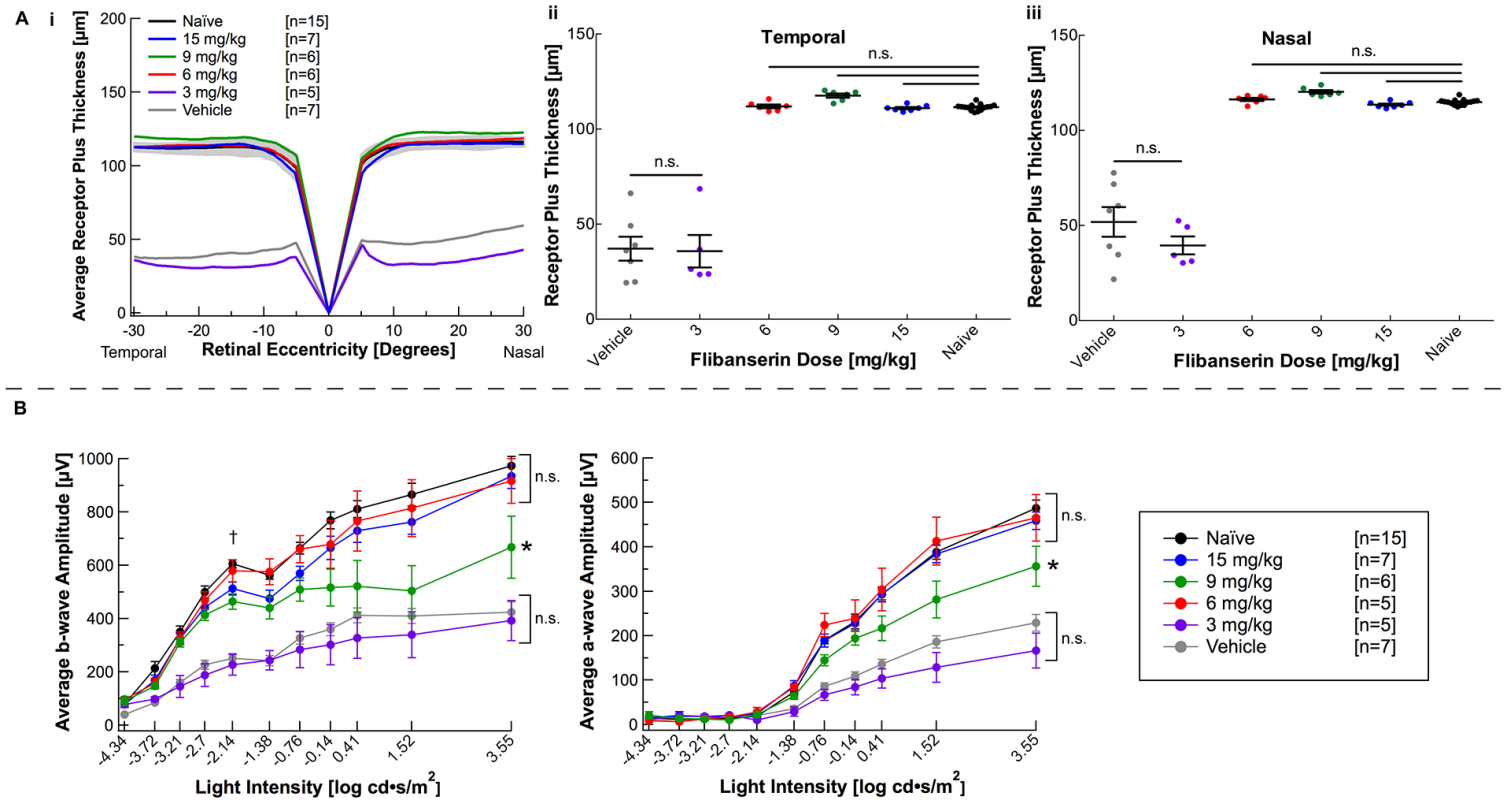
A one-day time course of flibanserin can preserve retinal morphology and function. **(A i)** A spider graph representing average right-eye receptor plus values demonstrates that a single dose of 6 mg/kg flibanserin or greater can significantly preserve retinal morphology to thicknesses comparable to naïve mice. The gray area indicates ± 2 SD of the naïve averaged data. (**A ii, iii**) Receptor plus thicknesses, with each dot representing average right and left eye thickness from one mouse, demonstrate that 6 mg/kg flibanserin provides morphological protection in the **(A ii)** temporal quadrant and the **(A iii)** nasal quadrant. Group averages are represented as mean ± standard error bar. **(B)** Mice treated with doses of 6 mg/kg and greater of flibanserin showed significantly higher ERG b-wave and a-wave responses at the highest flash intensity (3.55 log cd•s/m^2^) as compared to vehicle-treated mice. “†” indicates the greatest flash intensity that elicits a rod-only response (b_(max,rod)_). Group differences at the “†” flash intensity were statistically evaluated and are represented in S1B Fig. Individual ERGs were averaged with ERGs for mice within group and are represented as mean ± standard error. * indicates a significant difference from both the vehicle-treated group and the naïve group, *P* < 0.05. n.s. indicates non-significance with *P* > 0.05.

In order to ascertain whether flibanserin has the ability to act quickly and effectively, a one-day time course was evaluated. A single dose of 3, 6, 9 or 15 mg/kg flibanserin was injected immediately before light damage. Mice that received a 15, 9 or 6 mg/kg dose of flibanserin displayed complete morphological protection as assessed by OCT, and no statistical difference was found between their average REC+ thicknesses and those of naïve animals (Fig 3A, Table 1). Interestingly, a single injection of 3 mg/kg flibanserin failed to protect retinal morphology, as REC+ thickness was not statistically different from the vehicle-injected mice.

In regard to retinal function, all responses (b_max,rod_, b_max,rod+cone_, and a_max,rod+cone_) for the 6, 9 and 15 mg/kg flibanserin groups were statistically increased from the vehicle-treated group (Fig 3B, S1B Fig, Table 2). A single 3 mg/kg flibanserin injection failed to protect retinal function, thus corresponding with OCT results.

### Mechanism of Flibanserin-Mediated Neuroprotection

#### 5-HT_1A_ receptor-mediated neuroprotection

**Fig 4:**
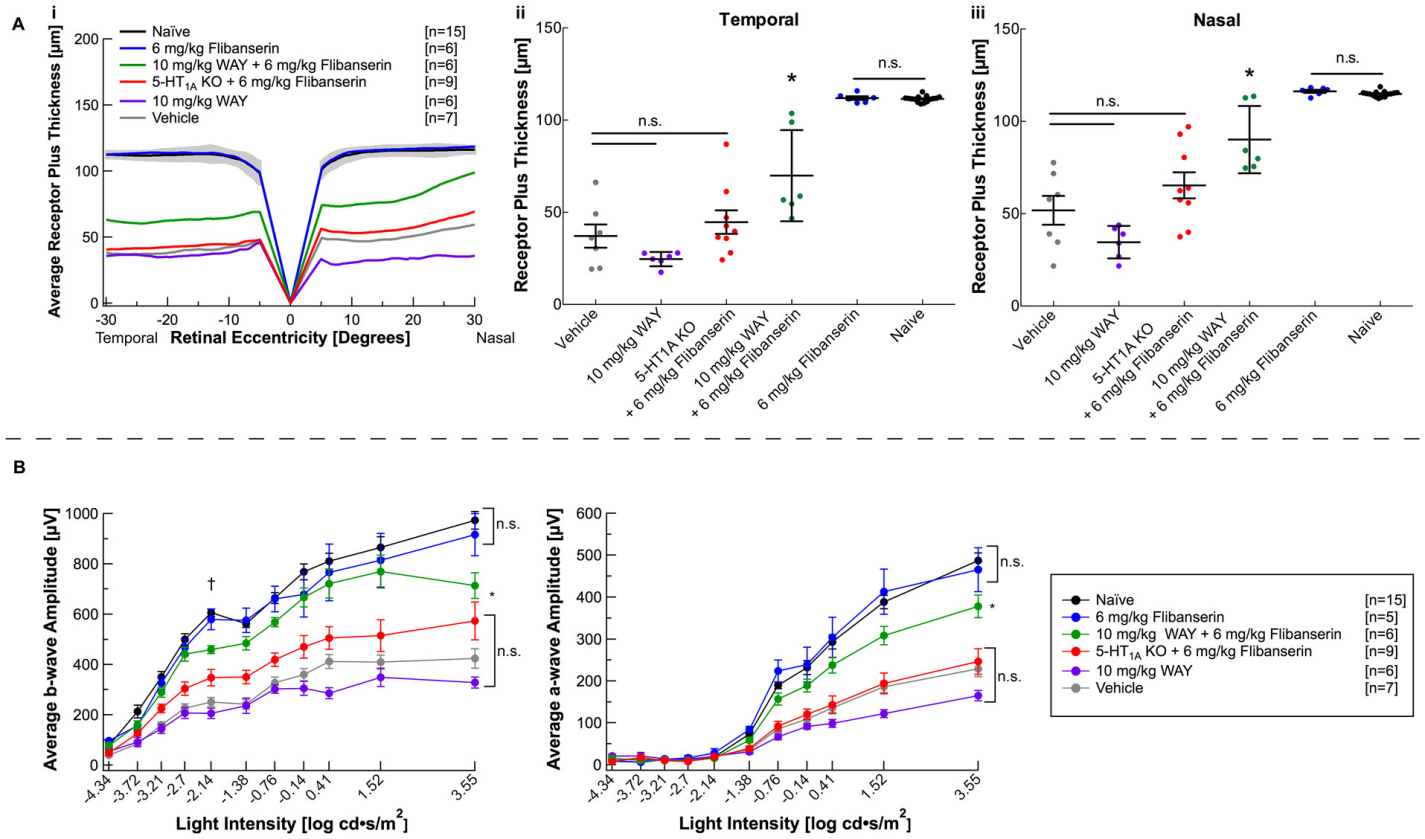
Flibanserin’s neuroprotective effects are 5-HT_1A_ receptor-mediated. (**A i**) A spider graph representing average right-eye receptor plus values demonstrates that pre-treatment with WAY 100635 prior to 6 mg/kg flibanserin (10 mg/kg WAY + 6 mg/kg Flibanserin, *green*) decreased average receptor plus thickness as compared to flibanserin-treatment without WAY 100635 (6 mg/kg Flibanserin, *blue*). A 6 mg/kg dose of flibanserin in 5-HT_1A_ knockout mice (5-HT1A KO + 6 mg/kg Flibanserin, *red*) resulted in average receptor plus thickness that was not significantly different from vehicle-injected and naïve mice. The gray area indicates ± 2 SD of the naïve averaged data. (**A ii, A iii**) Receptor plus thicknesses, with each dot representing average right and left eye thickness from one mouse, demonstrate that both pre-treatment of BALB/c mice with WAY 100635 prior to a flibanserin injection (10 mg/kg WAY + 6 mg/kg Flibanserin, *green*) and a flibanserin injection in 5-HT_1A_ knockout mice (5-HT1A KO + 6 mg/kg Flibanserin, *red*) significantly reduces the observed neuroprotective effects provided by a single 6 mg/kg dose of flibanserin (6 mg/kg Flibanserin, *blue*) in the **(A ii)** temporal quadrant and the **(A iii)** nasal quadrant. (**B**) Pre-treatment with WAY 100635 (10 mg/kg WAY + 6 mg/kg Flibanserin, *green*) mitigated the improvements to ERG a- and b-wave amplitudes provided by a single 6 mg/kg dose of flibanserin (*blue*). ERG a- and b-wave amplitudes were not improved by a single dose of 6 mg/kg flibanserin when administered to 5-HT_1A_ knockout mice (5-HT1A KO + 6 mg/kg Flibanserin, *red*). “†” indicates the flash intensity providing the b_(max,rod)_ value at which statistical analyses was also performed and presented in S1C Fig. * indicates a significant difference from both the vehicle-treated group and the naïve group, *P* < 0.05. n.s. indicates non-significance with *P* > 0.05.

Because flibanserin is both a 5-HT_1A_ agonist and a 5-HT_2A_ antagonist, both receptors may play a role in flibanserin-mediated neuroprotection. However, literature suggests that flibanserin’s mechanism of action is primarily due to stimulation of 5-HT_1A_ receptors. [33–39] To investigate the relative contribution of 5-HT_1A_ versus 5-HT_2A_ receptor occupancy in flibanserin-mediated neuroprotection, we utilized both a pharmacologic blockade of 5-HT_1A_ receptors and 5-HT_1A_ knockout mice. Pharmacologic blockade with 10 mg/kg WAY 100635, a 5-HT_1A_ antagonist, was administered to BALB/c mice 30 minutes prior to a single dose of 6 mg/kg flibanserin. As previously observed, a single injection of 6 mg/kg flibanserin in BALB/c mice preserved REC+ thickness (Figs 3A and 4A) and ERG a- and b-wave responses to the level of naïve mice (Figs 3B and 4B, S1B Fig). Pre-treatment with 10 mg/kg WAY 100635 prior to 6 mg/kg flibanserin resulted in a significant, albeit incomplete, reduction in REC+ thickness and ERG a- and b-wave values, as they were significantly less than naïve and 6 mg/kg flibanserin treated mice, but were significantly greater than vehicle-treated mice (Fig 4A and 4B, S1C Fig). To further delineate the necessity of 5-HT_1A_ in flibanserin-mediated neuroprotection, 6 mg/kg flibanserin was administered to 5-HT_1A_ knockout mice. In line with our pharmacologic findings, loss of 5-HT_1A_ almost completely negated the protective effect of flibanserin from light-induced retinopathy (Fig 4A and 4B, S1C Fig).

#### Augmentation of genes involved in cell survival

**Fig 5:**
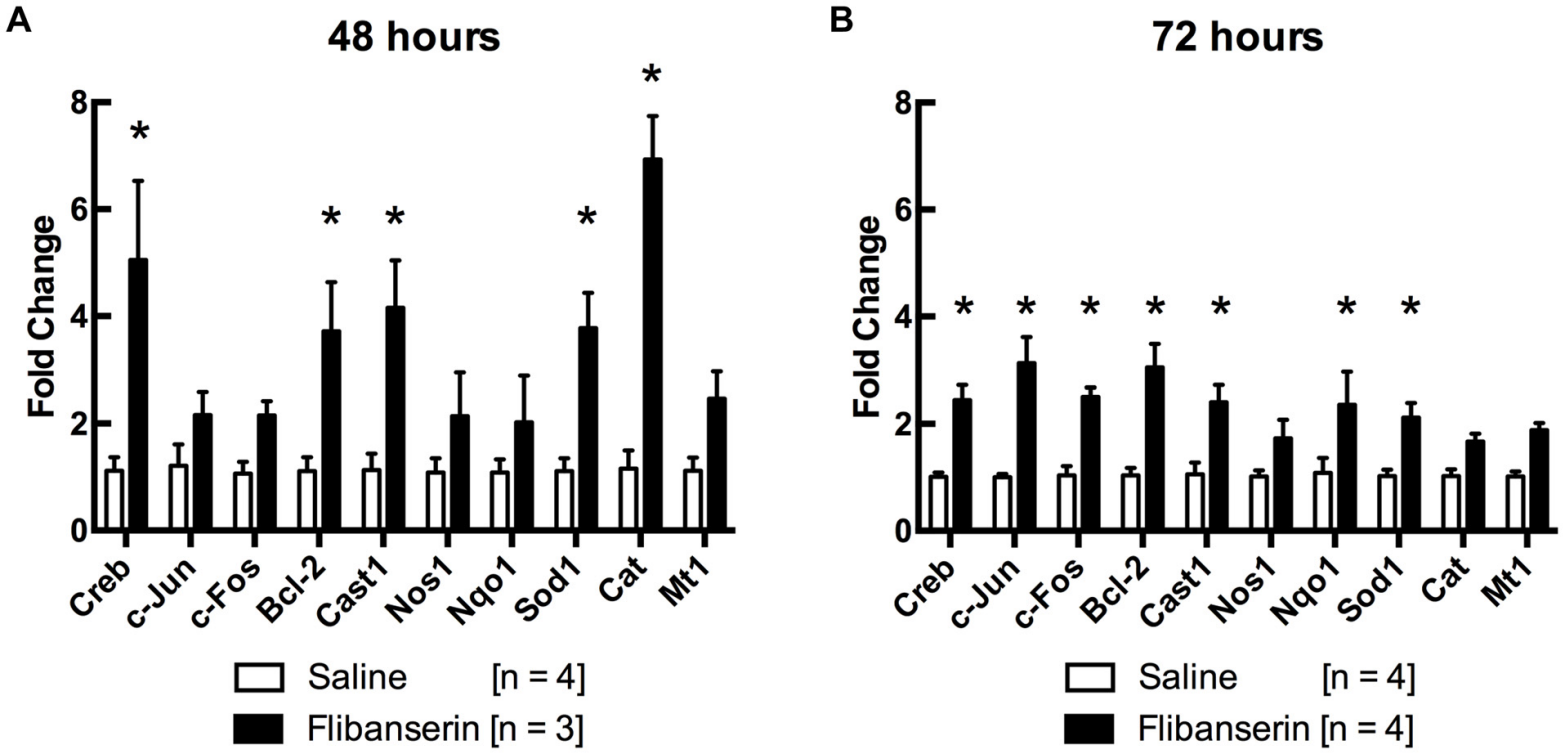
Flibanserin increases anti-apoptotic and antioxidant gene expression. **(A)** RT-qPCR shows that 48 hours following light-exposure, expression of *Creb*, *Bcl-2*, *Cast1*, *Sod1*, and *Cat* are significantly increased in flibanserin-injected mice versus vehicle-injected mice. **(B)** After 72 hours, expression of *Creb*, *c-Jun*, *c-Fos*, *Bcl-2*, *Cast1*, *Nqo1*, and *Sod1* are significantly increased in flibanserin-injected mice versus vehicle-injected mice. Analysis was performed using the ΔΔCt method with β-actin as the internal control. Significance was determined using a multiple t-test analysis (* indicates *P* < 0.05). cAMP Response Binding-element Protein (*Creb*), Cyclin D1 (*Cd1*), B-cell lymphoma 2 (*Bcl-2*), Calpastatin (*Cast1*), Nitric Oxide Synthase (*Nos1*), NAD(P)H quinone dehydrogenase 1 (*Nqo1*), Superoxide Dismutase 1 (*Sod1*), Catalase (*Cat*), Metallothionein 1 (*Mt1*).

Potential cell survival mechanisms involved in flibanserin-mediated neuroprotection were assessed via reverse transcription-quantitative PCR (RT-qPCR); specifically, genes involved with the inhibition of apoptosis or reduction of reactive oxygen species were evaluated. At 48-hours post-light exposure, flibanserin treatment significantly increased gene expression of cAMP response element-binding protein (*Creb*, 5.0-fold), B-cell lymphoma 2 (*Bcl-2*, 3.7-fold), Calpastatin 1 (*Cast1*, 4.2-fold), Superoxide dismutase 1 (*Sod1*, 3.8-fold), and Catalase (*Cat*, 6.9-fold) compared to vehicle treatment (Fig 5A). Similarly, 72-hours post-light exposure, flibanserin significantly increased the gene expression of *Creb* (2.4-fold), *Bcl-2* (3.0-fold), *Cast1* (2.4-fold), and *Sod1* (2.1-fold) versus vehicle (Fig 5B). Additionally, *c-Jun* (3.1-fold), *c-Fos* (2.5-fold), and NAD(P)H quinone dehydrogenase 1 (*Nqo1*, 2.4-fold) were significantly increased in flibanserin-injected mice compared to vehicle-injected mice.

## Discussion

This research demonstrates the novel finding that flibanserin, a dual serotonin receptor agonist and antagonist recently approved by the United States Food and Drug Administration for female HSDD, can completely protect the structural and functional integrity of albino BALB/c mouse retinas from light-induced retinopathy. Flibanserin’s neuroprotective effects began with a 3 mg/kg dose following a five-day time course and a 6 mg/kg dose following a one-day time course. A 15 mg/kg dose of flibanserin, following either time course, fully prevented the photoreceptor degeneration caused by bright light exposure.

Filbanserin demonstrated neuroprotection in a dose-dependent manner, consistent with the known pharmacokinetics of the drug. Borsini et al. showed that in the cortex, the 20% minimum receptor occupancy required for 5-HT_1A_ receptor activation was reached after a single 1 mg/kg dose, but that a 10 mg/kg dose was required to reach the same level of receptor occupancy in the hippocampus and dorsal raphe. [34] The dose needed to occupy receptors in the retina is currently unknown, but based on the finding that a single administration of 6 mg/kg or more was required to achieve neuroprotection, we hypothesize that the occupancy of 5-HT_1A_ receptors needed for neuroprotection in the retina is closer to that of the hippocampus and dorsal raphe.

In humans, three daily-administrations of flibanserin are required for the drug to reach steady state plasma concentrations. [33] The time to reach steady state concentrations of flibanserin is currently unknown in mice. However, due to flibanserin’s differing half-life in humans (11 hours) versus rats (0.9 to 1.9 hours), the time to reach steady state concentrations in rodents likely takes longer than three days. [34] Since flibanserin was only injected three times prior to light damage, maximum plasma and retinal concentrations may not have been reached with lower doses. Therefore, we hypothesize that if the number of daily-injections prior to light damage was increased, the total concentration of flibanserin would also increase, subsequently enhancing the neuroprotective effects of even the low doses.

Flibanserin has high affinity for the 5-HT_1A_ receptor (K_i_ = 1 nM) and a lower affinity for the 5-HT_2A_ receptor (K_i_ = 49nM), but lacks significant affinity for all other serotonergic and adrenergic receptors, allowing for the characterization of the neuroprotective targets, 5-HT_1A_ and 5-HT_2A_, in combination. [15–20, 22–26, 34] Flibanserin does, however, have moderate affinity for the dopaminergic D_4_ receptor (K_i_ = 4–24 nm), where it acts as an antagonist at low doses, and a partial agonist at higher doses. [34] It has been reported that D_4_ receptor agonists can inhibit oxidative stress-induced nerve cell death and protect against hypoxia/reoxygenation-induced cell death in cultured HT22 cells, which suggests that activation of the D_4_ receptor could be neuroprotective. [52, 53] Although D_4_ receptors have been reported in rodent retina, the threshold at which flibanserin begins functioning as a dopaminergic agonist is not well described in the mouse. [34, 54] Taken together, we considered the possibility that flibanserin-mediated neuroprotection could be due to D_4_ receptor activation, but our results suggested otherwise. WAY 100635, a 5-HT_1A_ antagonist and a potent D_4_ agonist, was tested in our light-induced retinopathy model, as it binds the D_4_ receptor with similar affinity (K_i_ = 16) as flibanserin. [34, 47] WAY 100635 failed to protect BALB/c retinas, both structurally and functionally, from light-induced retinopathy when given at a molar equivalent dose of the most effective dose of flibanserin, 15 mg/kg (Fig 4A and 4B, S1C Fig). Because this degree of D_4_ receptor activation did not protect the retina, it suggests that the observed neuroprotective effects of flibanserin are not likely mediated through D_4_ receptors.

In HSDD, Flibanserin is thought to mediate its effect through modulation of serotonin, dopamine and norepinephrine levels in brain regions involved in activating dopaminergic reward and sexual cue integration. [33, 35] Several studies suggest that Flibanserin’s action in the brain occurs through 5-HT_1A_ serotonin receptors. [34, 38, 39] This is supported by the finding that WAY 100635, a selective 5-HT_1A_ antagonist, completely antagonized the effects of flibanserin on serotonin, dopamine and noradrenaline in the rat prefrontal cortex. [35] Our results similarly suggest that flibanserin’s neuroprotective effects on the retina are mediated through the 5-HT_1A_ receptor. A single 10 mg/kg dose of WAY 100635 injected 30 minutes prior to a single 6 mg/kg injection of flibanserin severely reduced observed neuroprotective effects. In addition, 6 mg/kg of flibanserin failed to protect the retina from light-induced retinopathy in 5-HT_1A_ knockout mice. Whether flibanserin elicits any effect via the 5-HT_2A_ receptor remains to be elucidated.

The signaling cascade involved in 5-HT_1A_-mediated neuroprotection has yet to be fully characterized, therefore we performed gene expression assays to elicit the mechanisms responsible for flibanserin-mediated neuroprotection. The cell death signaling implicated in light-induced retinopathy involves the activation of nitric oxide synthase (*NOS*), increased intracellular calcium, disturbed mitochondrial function, and generation of oxidative stress, all of which can play a role in photoreceptor cell death in inherited retinal degeneration. [40, 55, 56] RT-qPCR results suggest that flibanserin provides neuroprotection by addressing the latter two components. In regard to mitochondrial dysfunction, the upregulation of Calpastatin (*Cast1*) and B-cell lymphoma 2 (*Bcl-2*) may lead to increased cell survival. Calpastatin is an endogenous inhibitor of calpains, which have been linked to caspase-dependent and caspase-independent cell death. [40, 57, 58] Arroba et al. demonstrated that maintained *Cast1* expression, via treatment with insulin-like growth factor-I (IGF-I), can significantly reduce apoptosis. [59] This effect was shown in both 661W cone cells and C57BL/6 mouse retinal explants grown in the presence of excess Ca^2+^, as well as in naïve rd1 mouse retinal explants. [59] *Bcl-2* has been described as an inhibitor of mitochondrial dysfunction during programmed cell death. [60] Additionally, transgenic expression of *Bcl-2* under control of the rhodopsin promoter has been shown to provide retinal neuroprotection in a light-induced retinopathy model, *rd1* mice, and S334ter-*Rho* mice. [61, 62]

Mitochondrial dysfunction often leads to oxidative stress, which occurs when reactive oxygen species capacity outweighs the antioxidant defense capacity. [63] Accordingly, antioxidants have been evaluated in several light-induced and inherited retinal dystrophy models, and have established their worth as neuroprotective agents. [17, 64–68] Flibanserin’s ability to upregulate antioxidant genes may play a significant role in cell survival. [40, 69] We have shown that flibanserin increases expression of Superoxide dismutase 1 (*Sod1*), Catalase (*Cat*), and NAD(P)H quinone dehydrogenase 1 (*Nqo1*). *Sod1* is an antioxidant responsible for the decomposition of free superoxide radicals to molecular oxygen and hydrogen peroxide, while *Cat* reduces the membrane-permeable hydrogen peroxide to water. [70, 71] *Nqo1*, activated by the transcription factor Nrf2, is responsible for quinone detoxification. [72–74] *Nqo1* operates by reducing ROS-generating quinones to hydroquinones, and has been reported to scavenge superoxides directly. [72, 75, 76] All three of these antioxidants have been able to provide protection against oxidative stress. For example, exogenous *Sod1* and *Cat* provided protection against retinal ischemic injury following increased intraocular pressure in different *in vivo* models. [77, 78] Adenovirus-mediated delivery of *Cat* was able to protect RPE cells from hydrogen peroxide-induced oxidative stress, as well as photoreceptors in an *in vivo* light damage model. [71] Additionally, *Nqo1* has played a prominent role in neuroprotection against oxidative stress in neuronal HT22 cells. [79]

Flibanserin treatment also led to significant increases in *c-Jun* and *c-Fos*. As a heterodimer, these proteins assemble into activator protein complex-1 (AP1), a transcription factor closely associated with apoptosis. [80] However, the role of the *c-Fos*/*c-Jun* heterodimer in apoptosis is complex. [80–82] While studies have shown that light-induced retinopathy can be prevented by the ablation of *c-fos*,[40, 56] this phenomenon only occurs in mice with a methionine substitution at RPE65 codon 450 and not in BALB/c mice which have a leucine mutation at this position. [82] Knocking out *c-fos* also did not prevent cell death in *rd1* mice. [56] Contrastingly, *c-Fos* and *c-Jun* have also been demonstrated to be anti-apoptotic. Gu et al. reported an absence of AP1 in dying photoreceptors, which argues against its role in photoreceptor apoptosis. AP1 activation was instead observed in Müller cells after light exposure, suggesting a pro-survival effect. [82]

In summary, when light-damage occurs, there is an increase of intracellular calcium, disruption of mitochondrial function, and oxidative stress. [40] We hypothesize that flibanserin treatment leads to the activation of *Creb*, which can upregulate *Cast1* and *Bcl-2* and protect against mitochondrial dysfunction. Furthermore, *Bcl-2* has the ability to modulate cellular calcium concentrations and increase antioxidant genes, such as *Sod1*, *Cat* and *NqO1*. [40, 60, 83–85] Although we saw a flibanserin-mediated increases in *c-Fos* and *c-Jun*, the role they play in neuroprotection remains unclear.

Flibanserin was recently approved by the FDA and marketed as Addyi™ for the treatment of HSDD and its side effects are relatively benign in both men and women; the most frequent adverse events being dizziness, somnolence, nausea and fatigue. [29] The potential to repurpose flibanserin for retinal neuroprotection is exciting. However, much work remains to transition it into a viable treatment for inherited retinal dystrophies, such as evaluation of its neuroprotective effects in an animal model of inherited retinal degeneration. Other avenues of delivery, such as intraocular injection, could also be explored to reduce possible systemic side effects. With its documented safety profile and FDA-approved status, further investigation of flibanserin for the treatment of retinal degenerations is warranted.

## Supporting Information

S1 Fig Statistical analyses of ERG b-wave responses at the b_(max, rod)_ response. **(A**) Five daily-doses of 3 mg/kg flibanserin and greater preserve the ERG b_(max, rod)_ response. **(B**) A single dose of 6 mg/kg flibanserin or greater can preserve the ERG b_(max, rod)_ response. **(C**) The ERG b_(max, rod)_ response observed after a single dose of 6 mg/kg flibanserin was significantly reduced in both 5-HT_1A_ knockout mice (5-HT1A KO + 6 mg/kg Flibanserin, *red*) and mice that received a pre-treatment of WAY 100635 (10 mg/kg WAY + 6 mg/kg Flibanserin, *green*). The averaged right and left eye data for each mouse is represented as a dot. Group averages are represented as mean ± standard error bar. * indicates a significant difference from both the vehicle-treated group and the naïve group, *P* < 0.05. n.s. indicates non-significance with *P* > 0.05.
(TIF)

S1 Table Primers for RT-qPCR. (DOCX)
