# Supplementary figures and images for: Retinal Neuroprotective Effects of Flibanserin, an FDA-Approved Dual Serotonin Receptor Agonist-Antagonist
Source: PLoS One. 2016 Jul 22;11(7):e0159776. doi: 10.1371/journal.pone.0159776 (PMC4957778; doi:10.1371/journal.pone.0159776)

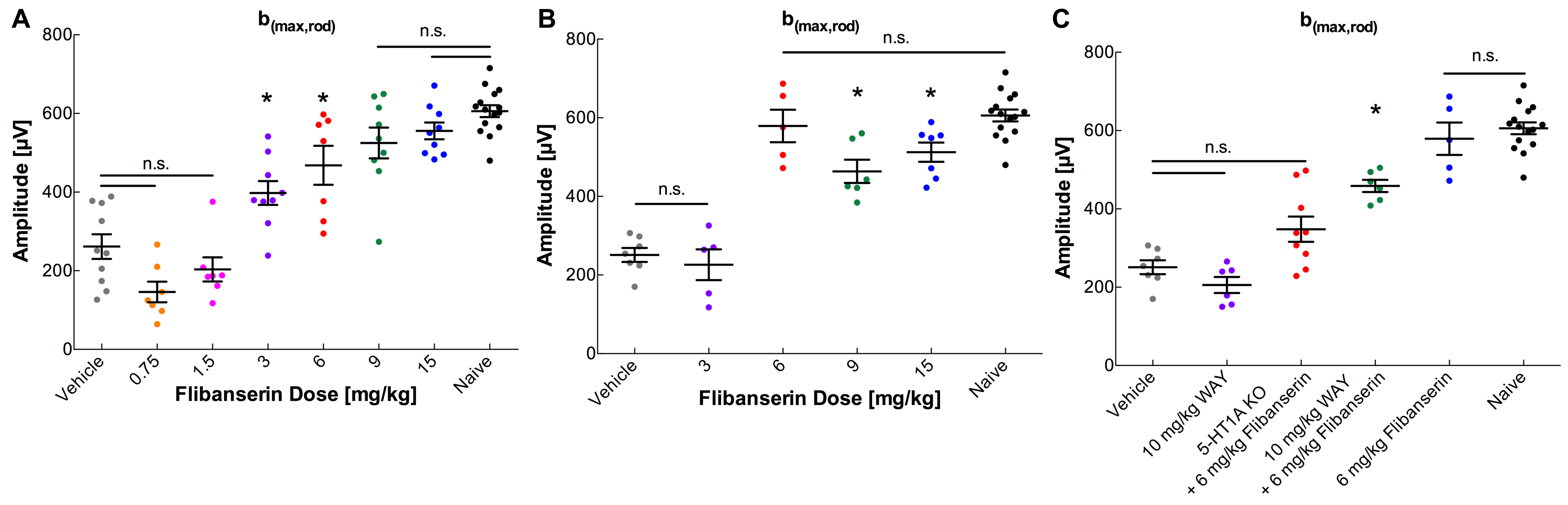

Supplement: S1 Fig — (A) Five daily-doses of 3 mg/kg flibanserin and greater preserve the ERG b(max, rod) response. (B) A single dose of 6 mg/kg flibanserin or greater can preserve the ERG b(max, rod) response. (C) The ERG b(max, rod) response observed after a single dose of 6 mg/kg flibanserin was significantly reduced in both 5-HT1A knockout mice (5-HT1A KO + 6 mg/kg Flibanserin, red) and mice that received a pre-treatment of WAY 100635 (10 mg/kg WAY + 6 mg/kg Flibanserin, green). The averaged right and left eye data for each mouse is represented as a dot. Group averages are represented as mean ± standard error bar. * indicates a significant difference from both the vehicle-treated group and the naïve group, P < 0.05. n.s. indicates non-significance with P > 0.05. (TIF) [file pone.0159776.s001.tif]
