# Supplementary material for: Retinal Neuroprotective Effects of Flibanserin, an FDA-Approved Dual Serotonin Receptor Agonist-Antagonist
Source: PLoS One. 2016 Jul 22;11(7):e0159776. doi: 10.1371/journal.pone.0159776 (PMC4957778; doi:10.1371/journal.pone.0159776)
Supplement: S1 Table — (DOCX) [file pone.0159776.s002.docx]

| Gene | Forward Primer | Reverse Primer |
| --- | --- | --- |
|  |  |  |
| *Creb* | TGGCTAACAATGGTACGGATG | TGTGCGGATCTGGTATGTTTG |
| *c-Jun* | GCAGAAAGTCATGAACCACG | AGTCCATCTTGTGTACCCTTG |
| *c-Fos* | TCCCCAAACTTCGACCATG | GCACTAGAGACGGACAGATC |
| *Bcl-2* | GAGCGTCAACAGGGAGATG | AATCCACTCACACCCCAAC |
| *Cast1* | AGAAAGGCAGGAGAAGTGTG | TGGCGTAGATGGTTTGTCTTG |
| *Nos1* | AGCAGAGATGAAAGACACAGG | CCCACAGATCATTGAAGACTCG |
| *Nqo1* | CGACAACGGTCCTTTCCAGA | CCAGACGGTTTCCAGACGTT |
| *Sod1* | TGCAGGGAACCATCCACTTCG | AACATGCCTCTCTTCATCCGC |
| *Cat* | CAGCGACCAGATGAAGCAGTG | GTACCACTCTCTCAGGAATCCG |
| *Mt1* | CCGGACTCGTCCAACGACTA | AGGAGCAGCAGCTCTTCTTG |
| β-actin | AGCGAGCATCCCCCAAAG TT | GGGCACGAAGGCTCATCATT |
|  |  |  |

cAMP response element-binding protein (*Creb*), B-cell lymphoma 2 (*Bcl-2*), Calpastatin 1 (*Cast1*), Nitric Oxide Synthase 1 (*Nos1*), NAD(P)H quinone dehydrogenase 1 (*Nqo1*), Superoxide dismutase 1 (*Sod1*), Catalase (*Cat*), Metallothionein 1 (*Mt1*)
